# Transilient Response to Acetone Gas Using the Interlocking p+n Field-Effect Transistor Circuit

**DOI:** 10.3390/s18061914

**Published:** 2018-06-12

**Authors:** Xinyuan Zhou, Jinxiao Wang, Zhou Wang, Yuzhi Bian, Ying Wang, Ning Han, Yunfa Chen

**Affiliations:** 1 State Key Laboratory of Multiphase Complex Systems, Institute of Process Engineering, Chinese Academy of Sciences, Beijing 100190, China; zhouxinyuan14@mails.ucas.edu.cn (X.Z.); lovekeyanzhou@163.com (Z.W.); bianyuzhi17@mails.ucas.edu.cn (Y.B.); wangying@ipe.ac.cn (Y.W.); 2 College of Chemical Engineering, University of Chinese Academy of Sciences, No. 19A Yuquan Road, Beijing 100049, China; 3 Center for Excellence in Regional Atmospheric Environment, Institute of Urban Environment, Chinese Academy of Sciences, Xiamen 361021, China; 4 College of Materials Science and Engineering, Xi’an Jiaotong University, Xi’an 710049, China; 13227885265@163.com

**Keywords:** Mn-doped ZnO, transilient response, field-effect transistor, diabetes, metal oxide semiconductor sensor

## Abstract

Low concentration acetone gas detection is significantly important for diabetes diagnosis as 1.8–10 ppm of acetone exists in exhaled breath from diabetes patients. A new interlocking p+n field-effect transistor (FET) circuit has been proposed for Mn-doped ZnO nanoparticles (MZO) to detect the acetone gas at low concentration, especially close to 1.8 ppm. It is noteworthy that MZO in this interlocking amplification circuit shows a low voltage signal of <0.3 V to the acetone <2 ppm while it displays a transilient response with voltage signal >4.0 V to >2 ppm acetone. In other words, the response to acetone from 1 ppm to 2 ppm increases by ~1233%, which is competent to separate diabetic patients from healthy people. Moreover, the response to 2 ppm acetone is hardly influenced by high relative humidity of 85%. In the meanwhile, MZO in this interlocking circuit possesses a high acetone selectivity compared to formaldehyde, acetaldehyde, toluene and ethanol, suggesting a promising technology for the widespread qualitative screening of diabetes. Importantly, this interlocking circuit is also applicable to other types of metal oxide semiconductor gas sensors. The resistance jump of p- and n-FETs induced by the change of their gate voltages is deemed to make this interlocking circuit produce the transilient response.

## 1. Introduction

Breath analysis has attracted much attention because it is a noninvasive method [1,2] compared with blood analysis and endoscopy. Hundreds of species of volatile organic compounds (VOCs) are in the exhaled gas of humans [3,4]. The concentrations of some specific VOCs are associated with abnormal medical conditions, including breast cancer [5], liver disease [6], lung cancer [7,8], and diabetes [9,10]. Clinical data show the concentration of acetone in exhaled gas from diabetes patients is in excess of 1.8 ppm while it is 0.3–0.9 ppm for healthy people [11,12]. Thus, many technologies have been developed to detect acetone for diabetes diagnosis, such as the selected ion flow tube mass spectrometry (SIFT-MS) [13,14] and gas chromatography-mass spectrometry (GC-MS) [15,16]. However, the challenge to the widespread application of these technologies is their large instrument size, complex operation and time-consuming process.

On the other hand, gas sensors based on metal oxide semiconductors (MOX), such as WO_3_ [17,18], In_2_O_3_ [19,20], SnO_2_ [21] and ZnO [22,23,24], have become promising candidates due to their trend of miniaturization, easy fabrication, low cost and high integration potential in portable devices [25,26]. The Gd-doped WO_3_/reduced graphene oxide nanocomposite [17] displayed improved gas sensing properties compared with the pure WO_3_, but it possessed a greater limit of detection (>2 ppm). Shen et al. [18] reported that Fe and C co-doped WO_3_ could detect the acetone level down to 0.2 ppm, but regrettably, the voltage signal of 2 ppm acetone is ~0.9 V while that of 0.9 ppm acetone is ~0.8 V, with the voltage signal increasing by only 13%, which tends to confuse diabetic patients and healthy controls. In addition, the poor selectivity of acetone restricted the further application of In_2_O_3_ nanobelts [19] and SnO_2_ nanostructures [21]. At the same time, the high relative humidity (~75%) considerably reduced the response of the Pt-decorated In_2_O_3_ nanoparticles to acetone at ppm-level [20]. Therefore, superior response and high selectivity toward the trace concentration acetone at high humidity (>80%) still remain great challenges in MOX gas sensors.

Our earlier reports [27,28,29] provided useful methods to enhance the response of MOX gas sensors. An n-type field effect transistor (FET) circuit has been proposed to amplify the apparent response of commercial sensor TGS 2602 (Figaro, Japan) [30] to toluene by around five times [27]. Besides this, the other commercial sensor, MP-4 sensor (Winsen, China) [31], in a coupling p+n FET circuit exhibits a ~14-fold higher apparent response to 150 ppm methane than that in the traditional circuit [29]. In this paper, a novel interlocking p+n FET amplification circuit is designed for Mn-doped ZnO nanoparticles (MZO) in order to detect acetone at low concentrations, especially at 2 ppm. The MZO in this interlocking p+n FET circuit shows a transilient response (voltage signal of 4.0 V) to 2 ppm acetone, whereas the response to the acetone lower than 2 ppm is negligible (voltage signal of <0.3 V). In other words, the response realizes a jump at the 2 ppm acetone, which is suitable for the qualitative detection of diabetes.

## 2. Design Scheme of the Amplification Circuit

**Figure 1:**
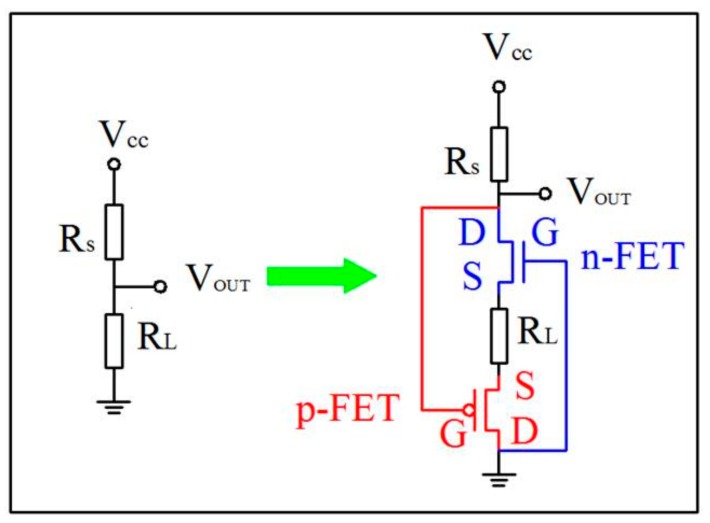
Design scheme of the traditional circuit and the interlocking p+n field effect transistor (FET) circuit for metal oxide (MOX) acetone sensor.

Figure 1 illustrates the circuit for MZO sensor which is developed from the traditional circuit to the interlocking p+n FET circuit. To begin with, the MZO sensor (R_S_) is connected in series with the load resistance (R_L_) when V_CC_ of 5 V is applied in the traditional circuit. The part voltage of R_L_ is the output voltage (V_OUT_). R_S_ of MZO (~95 MΩ) is much larger than R_L_ (~7 MΩ) to make the V_OUT_ as low as ~0.4 V, namely the baseline in air. When MZO is exposed to the acetone, R_S_ will decrease and the current will increase, inducing the growth of V_OUT_ in the acetone. Briefly, the change of R_S_ is the only factor to affect the change of V_OUT_ in the traditional circuit.

Then, an n-type and a p-type FETs are added into the circuit, forming the interlocking p+n circuit as shown in Figure 1. It is found that this circuit is different from the coupling or synergetic p+n circuits in Figure S1 in the supporting information according to our previous reports [28,29]. Similarly, the V_OUT_ is still low in air using the same R_L_ due to the negligible resistance of FET (R_FET_) at ON state (~100 Ω). Acetone exposure leads to the decrease of R_S_, followed by the growth of |V_GS_| of both FETs, making them work at OFF state (~1 GΩ). Ultimately, the V_OUT_ grows large dramatically. In short, the V_OUT_ is under the double influence of R_S_ and R_FET_ in the interlocking p+n FET circuit.

## 3. Experimental

The MZO acetone sensor is homemade as mentioned earlier [32]. The TGS 2602 and MP-4 sensors are commercial gas sensors used to detect toluene and methane, respectively. The 2SJ103 (TOSHIBA, Tokyo, Japan) [33] is a p-type transistor and the 2SK544 (SANYO, Moriguchi, Japan) [34] and 2SK427 (SANYO, Japan) [35] are the n-type transistors. The I_DS_–V_GS_ curves and I_DS_–V_DS_ curves of 2SK427 and 2SJ103 are obtained by the Keithley 4200 semiconductor analyzer (Lake shore cryotronics, Westerville, OH, USA). The sensing property of MZO is carried out on the static gas sensing test system Hanwei WS-30A (Winsen, Zhengzhou, China) [36,37,38] equipped with the load resistance card under the relative humidity, i.e., RH (25% and 85%), where RH is defined as the percentage of water vapor pressure in air versus saturated water vapor pressure at the same temperature. The p- and n-FETs purchased from the market are soldered onto the resistance card with D, S and G electrodes forming the interlocking p+n FET circuit as shown in Figure 1. Certain amounts of the 40 *v*/*v*% acetone solution were dropped with a micro syringe onto an evaporator in the test chamber (total volume 18 L) to generate different concentrations of acetone gas varying from 0.5 to 3 ppm. This method is also applicable for other gases, including formaldehyde, acetaldehyde, toluene and ethanol. All of the gases were tested at an operating temperature of 340 °C according to our previous report [32]. Then the commercial MP-4 and TGS 2602 MOX sensors were utilized to detect the methane and toluene by using the interlocking p+n FET circuit respectively. More details of their sensing property measurements are seen in the references [28,29]. For the traditional circuit and the interlocking p+n FET circuit, the voltage signal is defined as voltage difference in acetone and in air and the response time is defined as the time needed to reach the maximum voltage from baseline voltage.

## 4. Results and Discussion

### 4.1. Response and Selectivity to Acetone

The MZO material was synthesized by coprecipitation method as reported in our previous paper [32]. Typically, 50 mL ZnSO_4_ and MnSO_4_ aqueous solution was added drop-wisely into 100 mL NH_4_HCO_3_ solution, and the precipitation was rinsed and calcined at 500 °C for 2 h to get the product. The responses to acetone are measured from 200 °C to 370 °C, and the working temperature was optimized to be 340 °C and the doping concentration of Mn was optimized to be 2.2 mol %. In addition, the selectivity measurement illustrates that the response to acetone is much higher than those to ethanol, formaldehyde and benzene. This is due to the relatively higher surface acidity which prefers acetone adsorption and reaction. On the other side, if the surface is tuned to be more alkaline by CdO decoration, the response can be tuned to be higher to ethanol. In this study, only the MZO sensor is adopted for selective detection of acetone at the criteria concentration of 1–2 ppm for breath analysis. However, the voltage signals of MZO to 1 ppm and 2 ppm acetone are ~0.3 V and ~0.4 V, with the voltage signal increasing by only 0.1 V. Thus, it is essential to enhance the voltage signal of MZO to the trace concentration of acetone in order to diagnose diabetes.

**Figure 2:**
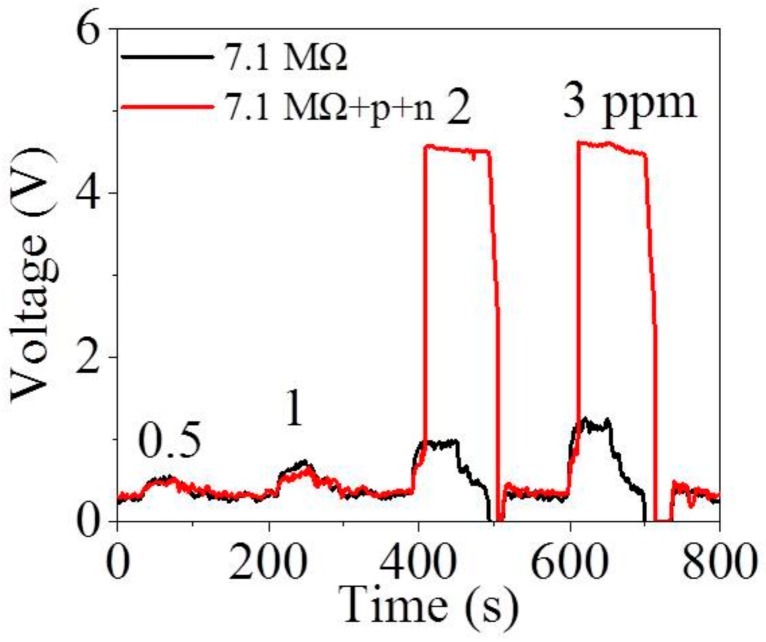
Output voltage of Mn-doped ZnO (MZO) to acetone from 0.5 to 3 ppm in the traditional electric circuit and the interlocking p+n FET circuit (R_L_ is 7.1 MΩ) under a humidity of 25%.

The output voltages of MZO sensor in the traditional electric circuit and the interlocking p+n FET circuit as a function of acetone concentrations between 0.5 and 3 ppm at 25% RH are shown in Figure 2. It is obvious that MZO sensors in both the interlocking p+n FET and the traditional circuits exhibit an almost equally small voltage signal (<0.3 V) to 0.5 and 1 ppm acetone while the former have much higher voltage signals (>4.0 V) to 2 and 3 ppm acetone than the latter (~0.5 V). It is found that the interlocking p+n FET circuit makes MZO realize a response jump at 2 ppm acetone, and thus the 2 ppm is named as the jump point of acetone. Figure 2 also illustrates that the output voltage of the traditional circuit has recovered to baseline when air was reinjected into the gas chamber. However, the V_OUT_ of the interlocking p+n FET circuit still remains at ~4.5 V for a long time. To recover the baseline, the power is turned off for ~5 s and then turned on again.

**Figure 3:**
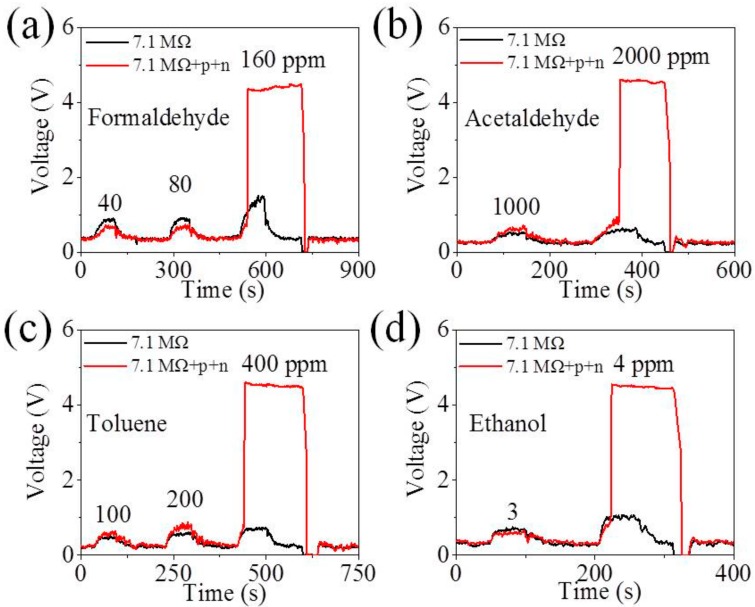
Output voltage of Mn-doped ZnO (MZO) to (**a**) formaldehyde from 40 to 160 ppm and (**b**) acetaldehyde from 1000 to 2000 ppm; (**c**) toluene from 100 to 400 ppm (**d**) ethanol from 3 to 4 ppm in the traditional circuit and the interlocking p+n FET circuit (R_L_ is 7.1 MΩ) under humidity of 25%.

The selectivity of the MZO sensor is taken into consideration. The response of MZO sensors to other species of VOCs, including formaldehyde, acetaldehyde, toluene and ethanol, is shown in Figure 3a–d. Taking the formaldehyde gas, for instance, the MZO sensor in this interlocking p+n FET circuit displays a transilient response (voltage signal of ~4.0 V) to 160 ppm formaldehyde while it responds a little (voltage signal of ~0.5 V) to formaldehyde lower than 160 ppm in Figure 3a. Other species of VOCs share the same feature. Figure 3a–d shows 160 ppm, 2000 ppm, 400 ppm and 4 ppm are the jump points of formaldehyde, acetaldehyde, toluene and ethanol, respectively, indicating that the MZO sensor in the interlocking p+n FETs circuit has excellent selectivity to acetone. Moreover, it is clear that the smallest gap exists between the jump points of ethanol and acetone, suggesting that the ethanol is likely to interfere with the acetone detection thus leading to the false diagnosis of the diabetes. Fortunately, many studies show that the ethanol level in the exhaled gas of healthy humans is found to be 770 ppb [39] or even lower [40,41], little affecting the acetone detection.

### 4.2. Acetone Detection under 85% Humidity

**Figure 4:**
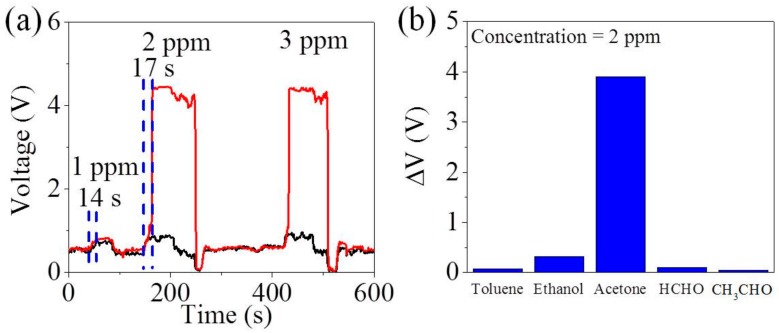
(**a**) Output voltage of MZO to acetone from 1 to 3 ppm in the traditional circuit and the interlocking p+n FET circuit (R_L_ is 7.1 MΩ) in the humid atmosphere of 85%; (**b**) The change of output voltage (ΔV) to different gases at 2 ppm (RH = 85%) for MZO in the interlocking p+n FET circuit (R_L_ is 7.1 MΩ).

More importantly, the relative humidity of the exhaled breath is higher than 80% RH at 1 atm and 25 °C [42], which has a negative effect on sensing performance such as water vapor poisoning [43]. Thus, we perform the gas detection of acetone under 85% humidity to investigate the potential feasibility of MZO sensors in the interlocking p+n FET for diagnosis of diabetes as shown in Figure 4a. In the present work, MZO sensors in the interlocking p+n FET are free from the influence of the high humidity and maintain a transilient response to 2 ppm acetone. In addition, the response to 2 ppm acetone is ~4.0 V while that of 1 ppm acetone is ~0.3 V. The response is increased by 3.7 V (1233%), much higher than those in the references [18,38,44,45], making it easy to sort out qualitatively diabetic patients from normal people. According to Figure 4a, the baseline voltage in air is ~0.5 V and the maximum voltage in 2 ppm acetone is ~4.5 V, therefore it needs a response time of ~17 s to change from ~0.5 V to ~4.5 V, faster compared with many published results [11,12,26,46]. In addition, ~340 °C is a common heating temperature for metal oxide MOX gas sensors to detect the acetone, which further diagnose the diabetes [10,26,46,47].

Similarly, five species gases (acetone, ethanol formaldehyde, acetaldehyde and toluene) at 2 ppm are tested to further prove the selectivity of MZO sensors in the interlocking p+n FET circuit at the relative humidity of 85% shown Figure 4b, where it is clear that the voltage signal (ΔV) of acetone is much higher than those of other species of gases. Besides, it is worthwhile to note that formaldehyde, a biomarker of breast cancer, is measured as 0.45–1.20 ppm exhaled from people suffering from breast cancer [5]. In addition, several VOCs including acetaldehyde and toluene appeared at levels of 10–20 ppb in normal people’s breath, whereas they are elevated to 10–100 ppb for lung cancer patients [7,8,48]. Therefore, diabetes diagnosis using MZO in this interlocking p+n FET circuit is able to exclude interference from breast cancer and lung cancer.

As to the expense, the cost increased by the two FETs in this interlocking circuit is only around 10% of the gas sensor. Moreover, this interlocking circuit enjoys the flexibility in the matching of p- and n-FETs compared to the synergetic p+n FET circuit with the restriction on the working curves of the FET [28]. In the meanwhile, MZO sensors in the interlocking p+n FET cannot be sensitive to the gas exhaled by the normal people. On the contrary, it outputs a high voltage signal (~4.0 V) exposed to the gas exhaled by the diabetes patients (generally 1.8–10 ppm [49]), in favor of the widespread and low cost screening of the diabetes.

### 4.3. Universality of the Interlocking p+n FET Circuit

To further prove the universality of this interlocking p+n FET circuit, the commercial MOX sensors MP-4 (Winsen, China) [31] and TGS 2602 (Figaro, Osaka, Japan) [30] are carried on the gas sensing property measurement at the relative humidity of 25%.

**Figure 5:**
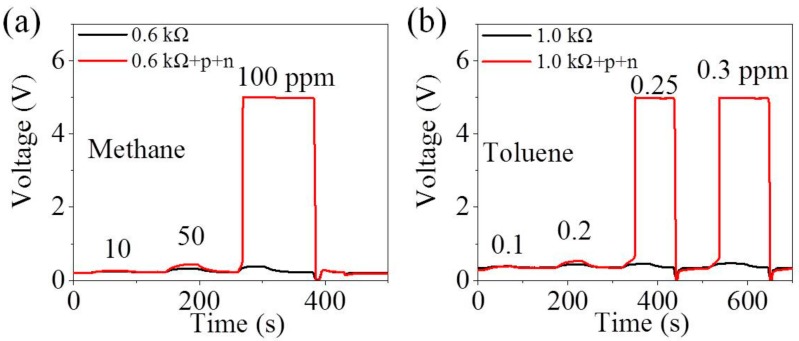
Output voltage of (**a**) MP-4 to methane from 10 to 100 ppm and (**b**) TGS 2602 to toluene from 0.1 to 0.3 ppm in the traditional circuit and the interlocking p+n FET circuit under humidity of 25%.

Figure 5a shows that 100 ppm is the jump point of methane when MP-4 sensors are placed in the interlocking circuit. As we all know, the lower explosion limit (LEL) of methane is approximately 50,000 ppm [50], and hence, MP-4 sensors can be used to detect the trace concentration of methane to avoid accidental explosion. Figure 5b illustrates that TGS 2602 sensor in this interlocking circuit produces the transilient response to 0.25 ppm toluene while it exhibits a tiny response to toluene lower than 0.25 ppm. According to the vehicle indoor air quality (VIAQ), 1.0 mg/m^3^ (0.26 ppm) is the maximum permitted limit of toluene in indoor air of a car. Thus, this p+n FET circuit entitles TGS 2602 to detect whether the concentration of toluene is beyond this standard value. In all, this interlocking p+n FET circuit is extensively applicable to MOX gas sensors.

### 4.4. Amplification Mechanism of the Interlocking p+n FET Circuit

In the earlier reports [27], the amplification effect of 2SK544 is due to the tiny change of the gate voltage inducing a dramatic resistance change of 2SK544. Thus, the mechanism of the interlocking p+n FET circuit is investigated from the perspective of the amplification effect of FET.

**Figure 6:**
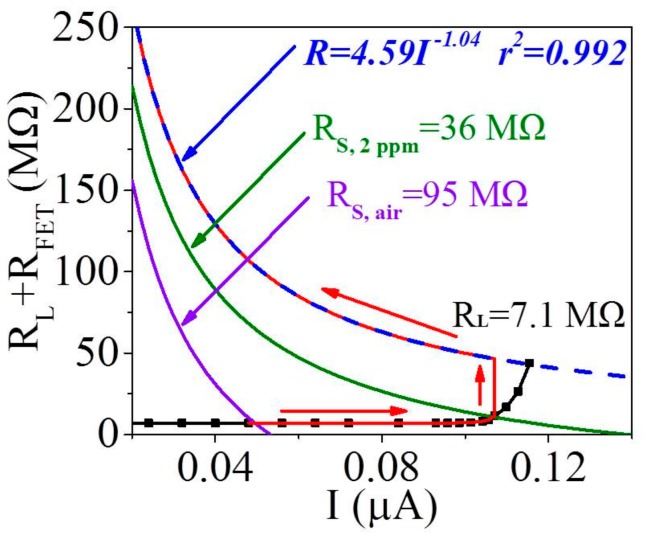
The approximate curve of interlocking p+n FET circuit with R_L_ of 7.1 MΩ (red solid curve). The blue dash curve is the fitting curve of the maximum points from five solid curves of n-type FET 2SK427 circuit with R_L_ 7.1, 10, 20, 30 and 50 MΩ respectively. And the fitting curve formula is R=4.59I^-1.04^ (r^2^=0.992)*.*

The black solid line in Figure 6 represents the curve of n-type FET amplification circuit with R_L_ of 7.1 MΩ. The blue dash line is the fitting curve of many maximum points from the curves of n-type FET amplification circuit using different R_L_, more details are seen in Figure S4. The violet, and green solid lines in Figure 6 are expressed respectively as

R_L_ + R_FET, air_ = V_CC_/I − R_S, air_ (1)

R_L_ + R_FET, 2 ppm_ = V_CC_/I − R_S, 2 ppm_ (2)

where V_CC_ is the applied voltage (5 V), R_FET, air_ and R_FET, 2 ppm_ represent FET resistance in air and 2 ppm acetone respectively. R_S, air_ and R_S, 2 ppm_ are the MZO sensor resistance (R_S_) in air and 2 ppm acetone individually. R_S, air_ and R_S, 2 ppm_ are experimentally measured as 95 MΩ and 36 MΩ respectively.

According to the interlocking p+n FET circuit in Figure 1, the following formulas can be obtained:

−V_GS(n)_ = I × R_L_ − V_DS(p)_ (3)

V_GS(p)_ = I × R_L_ + V_DS(n)_ (4)

V_OUT_ = −V_GS(n)_ + V_DS(n)_ (5)

where V_GS(p)_ and V_DS(p)_ are V_GS_ and V_DS_ of 2SJ103 respectively. V_GS(n)_ and V_DS(n)_ are V_GS_ and V_DS_ of 2SK427 respectively. I represents the circuit current.

The formula of the working curve of the interlocking p+n FET circuit can be obtained from Formulas (3)–(5).

R_FET_ = V_DS(n)_/I − V_DS(p)_/I (6)

V_DS(n)_ is under the double effect of V_GS_ of two FETs, and so is V_DS(p)_. Thus, both V_DS(n)_ and V_DS(p)_ are difficult to analyze and there is a tight interplay between 2SK427 and 2SJ103 in terms of the amplification effect, just as described in Formulas (3) and (4). Therefore, it is essential to simplify, approximate and estimate the working curve of the interlocking p+n FET circuit. This is divided into three stages:

- The first stage is in clean air. Both FETs work at ON state, so their resistances can be neglected compared to 7.1 MΩ. Therefore, this interlocking circuit in clean air is equivalent to the traditional circuit, denoted by the intersection of the black and violet solid lines seen in Figure 6;
- Introducing a trace concentration (<2 ppm) of acetone gas into the gas chamber is the second stage. The limited V_OUT_ amplification is generated by 2SK427 while 2SJ103 is still at ON state. Thus, this interlocking p+n FET circuit is equivalent to the single n-type FET circuit, i.e., R_L_ + R_FET_ = (V_DS(n)_ +V_GS(n)_)/I, whose curve overlaps the curve of the single n-type FET circuit with R_L_ of 7.1 MΩ (black solid line) shown in Figure 6;
- More than 2 ppm acetone gas is introduced in the third stage. Both 2SK427 and 2SJ103 tend to work at OFF state, whose resistances can arrive at 10 GΩ level. This stage is quite complex, thus the starting and ending points of this stage need to be determined for the sake of simplicity. It is obvious that the starting point is exactly the intersection (0.11 μA, 11 MΩ) of both the black and green lines in Figure 6. As to the ending point, its calculation is elaborated in the following part.

At the ending point of the third stage, two FETs are hypothesized to be at OFF state, so the circuit current decreases by 2–3 orders of magnitude. Therefore, Formulas (3) and (4) are simplified as below:

V_GS(n)_ = V_DS(p)_ (7)

V_GS(p)_ = V_DS(n)_ (8)

On the basis of Formulas (7) and (8) and V_DS_-I_DS_ curves of n-type 2SK427 and p-type 2SJ103 in Figure S2, V_GS(n)_, V_DS(n)_, V_GS(p)_ and V_DS(p)_ is calculated as −1.3 V, 3.7 V, 3.7 V and −1.3 V individually. The threshold voltages (V_T_) of 2SK427 and 2SJ103 are -0.6 V and 1.2 V respectively according to Figure S3, and hence V_GS(n)_ of −1.3 V and V_GS(p)_ of 3.7 V are large enough to turn of the FET to support reliability of hypothesis above. Furthermore, the minimum circuit current (I_min_) is calculated as 1.7 × 10^−4^ μA from the point where V_GS(n)_ = −1.3 V and V_DS(n)_ = 3.7 V in Figure S2a. Then I_min_ is substituted in the formula of blue dash line in Figure 6, the maximum resistance (R_max_) is calculated as 3.9 × 10^4^ MΩ. Therefore, the ending point of the third stage is denoted by the point (I_min_, R_max_), i.e., (1.7 × 10^−4^ μA, 3.9 × 10^4^ MΩ), not marked in Figure 6; more information is seen in Figure S4.

The working curve of the interlocking p+n FET circuit in the third stage is estimated. It starts with the point (0.11 μA, 11 MΩ) and then increases almost vertically, very similarly to the coupling p+n FET circuit previous [29], followed by arriving at the ending point (1.7 × 10^−4^ μA, 3.9 × 10^4^ MΩ) along the blue dash line. The working curve of this interlocking p+n FET circuit including three stages is the red solid line in Figure 6 and Figure S4, where the resistance jump of FET is the basic reason why this interlocking circuit can produce the transilient response.

**Figure 7:**
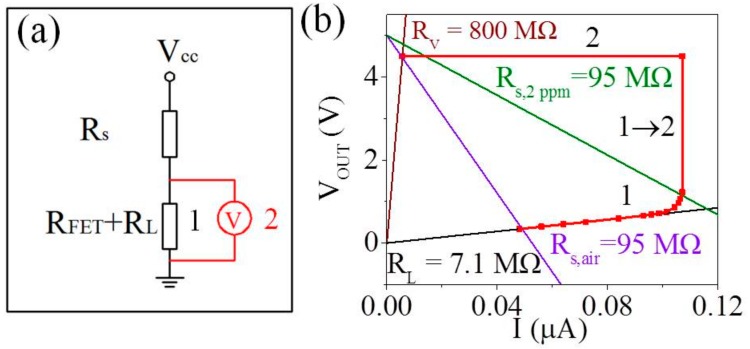
(**a**) Circuit schematic of the static gas sensing test system (**b**) The approximate output voltage of the interlocking p+n FET circuit with R_L_ of 7.1 MΩ (red solid curve).

It is worthwhile to note that the theoretical value of V_OUT_ is ~5 V (3.7 V + 1.3 V) according to Formula (5) while the actual value is ~4.5 V seen from Figure 2, Figure 3 and Figure 4, which can be explained by characteristics of the parallel circuit. From the circuit diagram in Figure 7a, R_FET_ and R_L_ are parallel with the voltmeter (~800 MΩ in Figure S5). R_FET_ and R_L_ in air are much smaller compared to the resistance of the voltmeter (R_V_), hence circuit 1 is closed in Figure 7a. However, both values of R_FET_ in 2 ppm acetone are much larger than R_V_. Thus, circuit 1 is open while circuit 2 is closed in Figure 7a and V_OUT_ is calculated to be ~4.5 V. Briefly, this interlocking p+n FET circuit can be switched between circuit 1 and 2, as shown in Figure 7b. Consequently, the interlocking p+n FET amplification circuit can serve as a novel technology for enhanced response to breath biomarkers and offer a potential platform for application in diabetes diagnosis.

## 5. Conclusions

In summary, a simple interlocking p+n FET circuit has successfully enhanced the response of MZO to acetone. It is interesting that a superior detecting capacity of MZO in this interlocking circuit with a transilient response (voltage signal of ~4.0 V) was obtained at ≥2 ppm acetone gas under high relative humidity of 85%, whereas it is trivial (~0.3 V) for acetone gas lower than 2 ppm. This response difference (~1233%) is high enough to satisfy the requirements for distinguishing diabetic patients from examinees. In addition, this technology also possesses a better acetone selectivity against formaldehyde, acetaldehyde, toluene and ethanol. Moreover, rapid response (~17 s) is appropriate to the breath analysis and the increased cost of this interlocking circuit is mainly the expense of the two FETs, about 10% of the gas sensor. Furthermore, this interlocking circuit can be directly utilized in other MOX gas sensors to detect the target gases. The transilient response of MOX sensor in the interlocking circuit is likely due to the jump of FET resistance induced by their gate voltage. These results demonstrate the promise for the qualitative detection of biomarker molecules in breath. Many efforts will be made to fabricate practical exhaled breath detecting sensors by employing the concepts in this study.

## Supplementary Materials

The following are available online at http://www.mdpi.com/1424-8220/18/6/1914/s1, Figure S1: The differences among the synergetic, coupling and interlocking p+n FET circuit for MOX acetone sensor, Figure S2: (a) V_DS_-I_DS_ curves of FET 2SK427, V_GS_ changes from −5 V to 0 V (step is 0.1 V). (b) V_DS_-I_DS_ curves of FET 2SJ103, V_GS_ changes from 0 V to 5 V (step is 0.1 V), Figure S3: I_DS_–V_GS_ curves of FETs 2SK427 (a) and 2SJ103 (b). (c) and (d) the calculation of V_T_, Figure S4: The approximate curve of interlocking p+n FET circuit with R_L_ of 7.1 MΩ (red solid curve). The blue dash curve is the fitting curve of the maximum points from five solid curves of n-type FET 2SK427 circuit with R_L_ 7.1, 10, 20, 30 and 50 MΩ respectively. The fitting curve formula is R = 4.59 I^−1.04^ (r^2^ = 0.992), Figure S5: (a) Output voltage of Mn doped ZnO (MZO) in the traditional circuit and the interlocking p+n FET circuit with R_L_ ON or OFF. (b) Circuit schematic of the static gas sensing test system.
