# Supplementary material for: Transilient Response to Acetone Gas Using the Interlocking p+n Field-Effect Transistor Circuit
Source: Sensors (Basel). 2018 Jun 12;18(6):1914. doi: 10.3390/s18061914 (PMC6021865; doi:10.3390/s18061914)
Supplement: Supplementary file 1 [file sensors-18-01914-s001.pdf]

sensors

Article

Supplementary Materials: Transilient Response to Acetone Gas using the Interlocking p+n Field-effect Transistor Circuit

Xinyuan Zhou[1,2,3], Jinxiao Wang [4], Zhou Wang[1], Yuzhi Bian[1,2], Ying Wang[1], Ning Han[1,3,*] and Yunfa Chen[1,3,*]

[1] State Key Laboratory of Multiphase Complex Systems, Institute of Process Engineering, Chinese Academy of Sciences, Beijing 100190, China

[2] University of Chinese Academy of Sciences, No. 19A Yuquan Road, Beijing 100049, China zhouxinyuan14@mails.ucas.edu.cn

[3] Center for Excellence in Regional Atmospheric Environment, Institute of Urban Environment, Chinese Academy of Sciences, Xiamen 361021, China

[4] College of Materials Science and Engineering, Xi'an Jiaotong University, Xi'an 710049, China

* Correspondence: nhan@ipe.ac.cn; chenyf@ipe.ac.cn; Tel.: +86-010-6255-8356

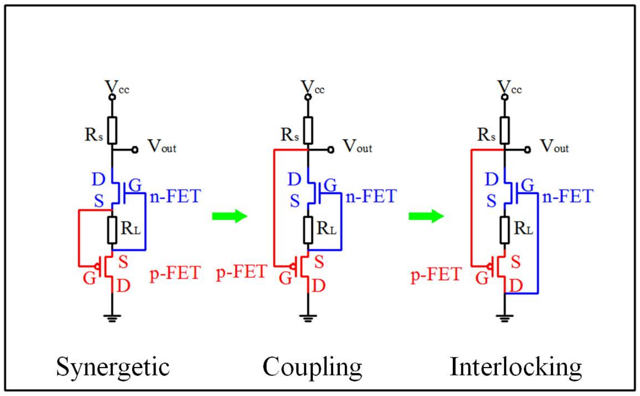

Figure S1. The difference among the synergetic, coupling and interlocking p+n FET circuit for MOX acetone sensor.

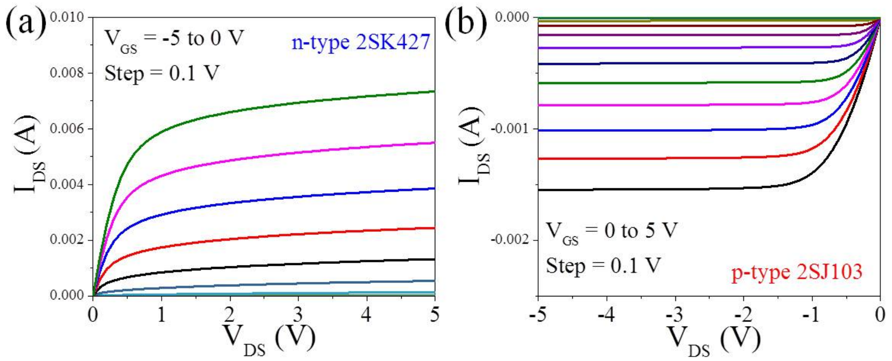

Figure S2. (a) V_{DS}-I_{DS} curves of FET 2SK427, V_{GS} changes from -5 V to 0 V (step is 0.1 V). (b) V_{DS}-I_{DS} curves of FET 2SJ103, V_{GS} changes from 0 V to 5 V (step is 0.1 V).

$$V_{GS(n)} = V_{DS(p)} \cdots\cdots\cdots (7)$$

$$V_{GS(p)} = V_{DS(n)} \cdots\cdots\cdots (8)$$

$$V_{GS(n)} = -1.3V \qquad (V_{T(n)} = -0.6V)$$

$$V_{GS(p)} = 3.7V \qquad (V_{T(p)} = 1.2V)$$

$$\mathrm{I}_{\min} = 1.65 \times 10^{-4} \mu A$$

$$V_{OUT} = IR_L - V_{DS(p)} + V_{DS(n)} = 5V$$

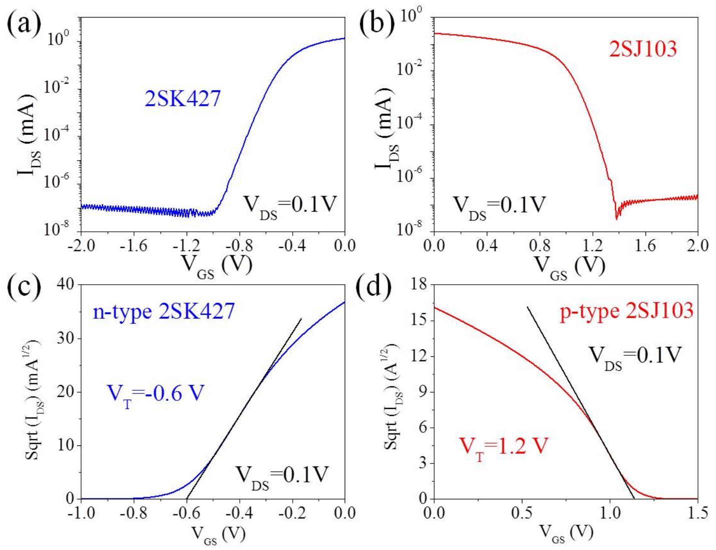

Figure S3. I_{DS}-V_{GS} curves of FETs 2SK427 (a) and 2SJ103 (b). (c) (d) the calculation of V_T.

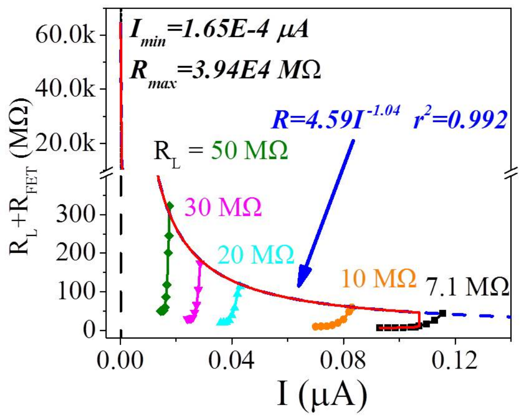

Figure S4. The approximate curve of interlocking p+n FET circuit with R_L of 7.1 MΩ (red solid curve). The blue dash curve is the fitting curve of the maximum points from five solid curves of n-type FET 2SK427 circuit with R_L 7.1, 10, 20, 30 and 50 MΩ respectively. The fitting curve formula is $R = 4.59\ I^{-1.04}$ (r^2=0.992).

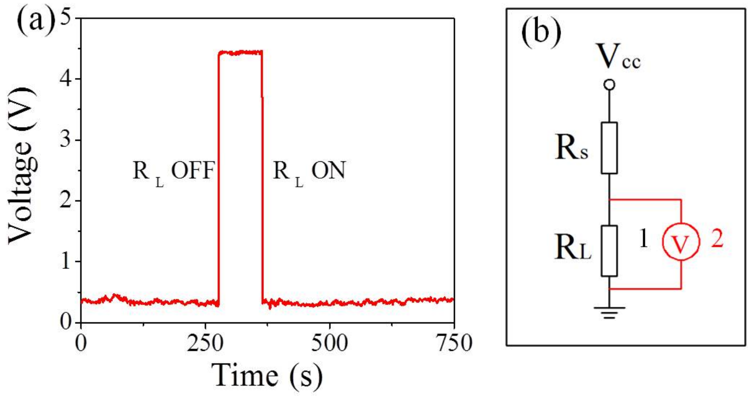

Figure S5. (a) Output voltage of Mn doped ZnO (MZO) in the traditional electric circuit and the interlocking p+n FET circuit with R_L ON or OFF. (b) Circuit schematic of the static gas sensing test system.

When R_L is connected, the circuit 1 is closed. When R_L is cut off, the circuit 1 is open, but the circuit 2 is closed and R_V is calculated to be ~ 800 MΩ.
